# Energy and New Economic Approach for Nearly Zero Energy Hotels

**DOI:** 10.3390/e21070639

**Published:** 2019-06-28

**Authors:** Francesco Nocera, Salvatore Giuffrida, Maria Rosa Trovato, Antonio Gagliano

**Affiliations:** 1 Department of Civil Engineering and Architecture, University of Catania, 95123 Catania, Italy; 2 Department of Electric and Computer Engineering, University of Catania, 95125 Catania, Italy

**Keywords:** nZEBs, hotels, energy, economic analysis

## Abstract

The paper addresses an important long-standing question in regards to the energy efficiency renovation of existing buildings, in this case hotels, towards nearly zero-energy (nZEBs) status. The renovation of existing hotels to achieve a nearly zero-energy (nZEBs) performance is one of the forefront goals of EU’s energy policy for 2050. The achievement of nZEBs target for hotels is necessary not only to comply with changing regulations and legislations, but also to foster competitiveness to secure new funding. Indeed, the nZEB hotel status allows for the reduction of operating costs and the increase of energy security, meeting the market and guests’ expectations. Actually, there is not a set national value of nZEBs for hotels to be attained, despite the fact that hotels are among the most energy-intensive buildings. This paper presents the case study of the energy retrofit of an existing historical hotel located in southern Italy (Syracuse) in order to achieve nZEBs status. Starting from the energy audit, the paper proposes a step-by-step approach to nZEBs performance, with a perspective on the costs, in order to identify the most effective energy solutions. Such an approach allows useful insights regarding energy and economic–financial strategies for achieving nZEBs standards to highlighted. Moreover, the results of this paper provide, to stakeholders, useful information for quantifying the technical convenience and economic profitability to reach an nZEBs target in order to prevent the expenses necessary by future energy retrofit programs.

## 1. Introduction

With the adoption of the Energy Performance of Buildings Directive in 2010, both the building industry and Member States (MSs) undertook new challenges [1]. In Italy, existing buildings represent the majority of the building stock and the largest and most cost-effective energy saving potential [2,3]. For this reason, an interesting challenge is the upgrading, of the existing building stock, to nearly zero-energy (nZEBs). The concept of nZEBs refers to a building with a net energy consumption of nearly zero over a typical year. The target of a nZEB is not limited to minimize the energy consumption with an energy efficient envelope and the rational use of energy (RUE), but requires balancing their energy requirements with the exploitation of on-site renewable sources, locally available, non-polluting, and low-cost [4].

Specifically, hotels represent a fascinating challenge since they are usually complex building systems and, at the same time, they need to minimize their energy costs without compromising the quality of their guests’ stay [5,6,7]. Hotel buildings have some special features that must be considered when planning an energy renovation: (a) Seasonal operation with a large fluctuation in energy demand and large use of delivered energy for non-hosting functions, such as spas, swimming pools, saunas, gyms, kitchens, laundry, etc., to ensure customers’ comfort and expectations [8]. Although no collective data are available on global energy consumption in the hotel sector, it is estimated that 97.5 TWh power was used in hotel facilities worldwide in 2001 [9]. Numerous researches on energy use in hotels in Europe show that hotels consume around 200–400 kWh/m^2^ per year and almost half of it (48%) is used for HVAC (Heating, ventilation, and air conditioning) [10]. Nevertheless, the wide variety of existing hotel buildings, which may differ in age, dimensions, and location, does not allow a unique approach to the energy retrofit. Therefore, the retrofit interventions must be carefully defined and designed, particularly in the case of historical or architectural constraints [11]. The hotels are among the most energy-intensive buildings, consuming large amounts of energy per unit surface with large use of fossil fuels. Use of renewable energy resources in the Mediterranean basin are not exploited enough and could be used for heat and power generation, replacing fossil fuels, offering a profit to the owner, and reducing carbon emissions, with resulting environmental benefits. Obviously, renewable energy technologies which can be used in hotels depends on the availability of the energy source. Some of them are abundant, like solar energy, and others are site-dependent, like wind energy and biomass [12]. However, the combined utilization of energy-saving technologies with renewable energy resources in hotels could minimize or zero the energy consumption as well as the carbon emissions due to the operational energy use in them [13,14].

Based on the case study of a small-to-medium size hotel, the authors have outlined different cost-effective energy retrofit strategies and measures. After that, they have developed an integrated assessment model [15,16], in order to evaluate not only the feasibility and environmental sustainability of retrofit solutions but, also, the opportunity to maximize the trade-off between technical–environmental and economic–financial performances [17,18].

## 2. Materials and Methods

### 2.1. Energy Saving Issues

In order to obtain the expected energy saving, the evaluation of a retrofit intervention requires the identification of the most effective solutions according to the particular conditions of the site and the type of building which must be renovated [4]. Therefore, it is essential to define a clear picture of the current state of the building. Consequently, in the first stage, a survey was carried out in order to collect reliable information concerning the HVAC systems, the lighting systems, the geometry of the building elements, their current condition, the pathologies affecting them, and the deficit to be addressed. Moreover, specific information on the features of the site, the climatic conditions, the orientation, and the current energy consumption was collected. All data regarding the building undergoing renovation was used for modelling a specific case study, in order to provide a reliable integrated assessment model. Thus, several scenarios characterized by different technological solutions and retrofitting strategies could be tested, both from the technical–environmental viewpoint and from the economic–financial perspective [19,20]. This multidimensional approach allowed for several factors to be kept under control that could influence the behavior of the refurbished building in terms of energy performance indexes (EP), energy classification (EC), and carbon dioxide reduction (ECO_2_). Moreover economic–financial indexes as net present value (NPV), internal rate of return (IRR), and discount payback period (DPbP) were calculated [21,22].

Since the above mentioned indices (i.e., EP, EC, CO_2_, NPV, IRR, DPbP), have different scales and measure units, a novel procedure was developed to encompass them into a single index expressed in a standard scale range. Such a procedure requires the homogenization of the diverse factors that influence the retrofit design according to the stakeholders (e.g., public authority and customers) and the stockholders (the entrepreneurs). Consequently, such a multidimensional approach provides the decision makers with broad information about the effects any change envisaged throughout the design path can have on the global result of the retrofit process

The global energy performance index for non renewable energy EP_gl,nr_ and for renewable energy EP_gl,rin_, the energy classification (EC) and CO_2_ emissions due to the building energy consumptions were calculated before and after the proposed retrofit actions. Analyses and simulations were performed using Termus Software, which complies with the following regulations: UNI EN ISO 15316 [23], UNI EN ISO 13790 [24], UNI TS 11300 parts 1–2 [25,26].

### 2.2. Economic–Finacial Valuation Issues

The economic–financial performances are measured carrying out a discounted cash flow analysis [27,28,29,30] by means of the following indices:

- Net present value, NPV, the sum of the discounted inflows and outflows over the whole lifespan T, at the discount rate r; it needs to be positive;
- Internal rate of return, IRR, the discount rate that makes NPV of all cash flows from the investment equal to zero; it needs to be greater than r, given that the latter is assumed as the global cost (interest rate and opportunity cost) of the invested capital;
- Discounted payback period, DPbP, is the time span over which the initial investment cost is recovered by the net future discounted cash flows.

The valuation-programming model consists of the following stages:

1. A set of approaching nZEB solutions $a_{i}\;\left(i=1,\;2,\;\ldots ,\;9\right)$ were designed and characterized from the perspectives of the above mentioned technical–environmental and economic–financial performances, respectively $T_{i}\;\left(i=1,\;2,\;\ldots ,\;4\right)$ and $E_{i}\;\left(i=1,\;2,\;\ldots ,\;3\right)$;
2. T and E were successively normalized in a standard scale of scores, $k_{T}=f\left(T\right)$, $k_{E}=f\left(E\right)$, ranging from 1 (lowest performance) to 5 (highest performance);
3. Two overall scores were attributed to each $a_{i}$: $K_{Ti}=\sum_{i=1}^{4}k_{Ti}\lambda_{Ti}$ and $K_{Ei}=\sum_{i=1}^{4}k_{Ei}\lambda_{Ei}$, where $\lambda_{Ti}$ and $\lambda_{Ei}$ are the weights measuring the relative importance of each performance of group T or E compared to the others in the same group, under the conditions: $\sum_{i=1}^{4}\lambda_{Ti}=1$ and $\sum_{i=1}^{3}\lambda_{Ei}=1$;
4. Based on scores $K_{Ti}$, the solutions $a_{i}$ are arranged in a ranking $R_{T\left(W\right)}$ depending on the chosen weight system W; as the weight system reflects the perspective and the prospects of the decision makers, we made some hypotheses about it supposing three different scenarios (displayed in Section 4.2);
5. A set $A_{w}$ of strategies $A_{wi}\;\left(i=1,\;2,\;\ldots ,\;9\right)$ was arranged; each $A_{wi}$ is a bundle of $a_{i}$ packed progressively including an increasing number of them, according to the above-mentioned ranking, so that: $A_{w1}$ includes only the best $a_{i}$; $A_{w2}$ includes the best two $a_{i}$; and so on to the last strategy $A_{w9}$ including all solutions; “the best” means the one(s) at the top of the above-mentioned ranking;
6. $K_{Ti}=f\left(W\right)$ and $A_{i}=f\left(W\right)$ as well, so that each set of strategies is associated to a weight system and given W, $A_{w}$, it follows that, within each $A_{w}$, the best economic–financial $A_{wi}$ can be selected based on $K_{Ei}$.

The input data are referred to as the technical–economic situation in Italy in 2017. According to the Italian scenario, the operating costs are referred to as the unit prices of natural gas (0.10 €/kWh) and electricity (0.25 €/kWh) [31]. The operational life and maintenance costs of the energy efficiency measures and their components are referred to as UNI EN 15459:2008 [32]. The government incentives are accounted for each solution [33]. The economic analysis is performed in nominal terms; the discount rate is referred to as the interest rate reported in [34] and prudentially assumed at 5% as the basis for the scenario analyses.

## 3. Case Study

### 3.1. Description 

**Figure 1:**
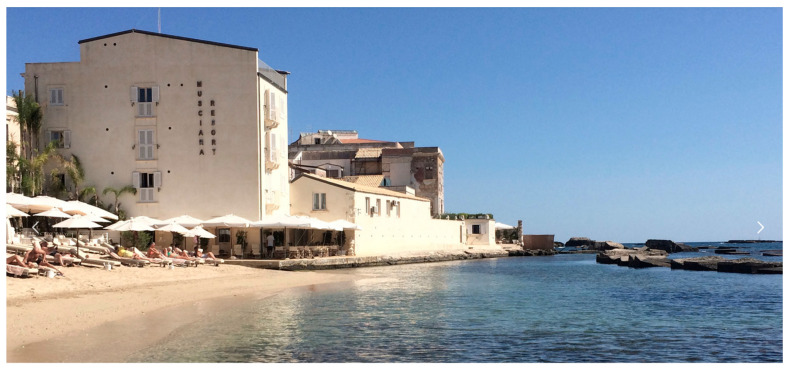
Views of the hotel’s shore and entrance.

The Hotel Musciara (Figure 1) is a coastal hotel located in Ortygia, the old town of Syracuse in southeastern Sicily, on an islet connected to the mainland by two bridges. Syracuse’s climate is classified as warm and temperate. In the winter, there is much more rainfall than in the summer. This location is classified as Csa by Köppen and Geiger. The average annual temperature is 17.8 °C. In a year, the average rainfall is 504 mm. Syracuse has 799 heating degree days (HDD) and belongs to Zone B according to the national climatic zoning.

The building, located on the northeastern water front and built at the beginning of the twentieth century, was refurbished and converted into a hotel about twelve years ago (2006). It is worth mentioning that, because of its historical features, the building was subject to some constraints to comply with during the renovation process. In particular, the main façade cannot be insulated from the outside, because of aesthetic reasons. The hotel has a rectangular plan, with six stories above ground level. Musciara Resort operates annually and comprises twelve guestrooms for two people only, each of them fully provided with appliances. The extra facilities offered by the resort are: Private beach, restaurant, outdoor restaurant, meeting room, laundry service, whirlpool, satellite TV, and hair dryer. The whole structure has a gross surface of 430.66 m^2^ and a gross volume of 1259 m^3^; the shape factor is 0.45 m^−1^.

### 3.2. Building Envelope and Energy Systems’ Features

The Hotel Musciara presents a very traditional structure with load bearing masonry walls (65 cm thick) without thermal insulation. The roof is clad with typical Sicilian tiles, has a pitch of 25°, and is insulated. The floors and the attic have a traditional timber bearing structure. The wooden floor (28 cm thick) of the attic is insulated. The windows are single pane and have a timber frame without thermal break, and they are provided with external wooden blinds. The U-values of the main envelope components, calculated after site inspection, are as follows:

⇒ load bearing masonry walls: U = 1.792 W·m^−2^·K^−1^
⇒ roof: U = 0.443 W·m^−2^·K^−1^
⇒ attic floor: U = 0.318 W·m^−2^·K^−1^
⇒ ground floor: U = 0.970 W·m^−2^·K^−1^
⇒ attic floor: U = 0.318 W·m^−2^·K^−1^
⇒ ground floor: U = 0.970 W·m^−2^·K^−1^
⇒ windows: U = 4.38 W·m^−2^·K^−1^

The building is heated and cooled by an air-to-water type heat pump model Daikin RXYQ14M9W1B, with 45 kW heating capacity and 40 kW cooling capacity [35]. The heat pump has a 13.40 kW nominal power input for heating and a 11.70 kW nominal power input for cooling [36,37]. Eighteen two-pipes fan coil units, placed in the false ceiling, are the terminals of the heating and cooling system. A furnace model Vaillant VK/1 Turbo VIT powered by natural gas (rated output 31.5 kW) is used for domestic hot water (DHW) production. The DHW loop also includes a 1500 L water storage tank. At present, the building does not have a mechanical ventilation system (except for exhaust air systems in bathrooms and kitchens) and it does not use any on-site renewable energy source. The temperature set-points for space heating and cooling were fixed, respectively, at 20 °C from 1 December to 31 March (heating period), and at 26 °C from 1 April to 30 November (cooling period). The infiltration rate is 0.5 air changes per hour. The hotel is classified in the category of three stars so the daily consumption of domestic heat water is set to 80 L/person/day. Occupancy levels of the different zones are fixed as follows: 10 m^2^/person for guestrooms, 0.2 m^2^/person for lobby and café, 0.6 m^2^/person for breakfast rooms, 0 m^2^/person for the corridors and service space. Equipment power densities are set equal to 6 W/m^2^.

**Figure 2:**
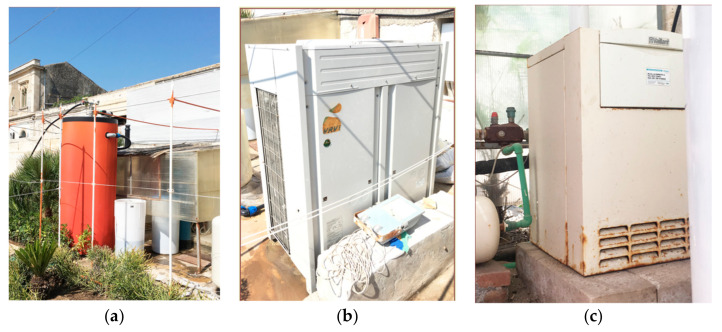
From left to right: (**a**) DHW system, (**b**) furnace, (**c**) heat pump.

Manual opening of the windows provides air change during daily cleaning. The lighting system consists of both incandescent and fluorescent lamps installed in rooms, corridors, and service areas. No control systems are installed. In Figure 2, DHW system, furnace and heat pump are reported.

### 3.3. Current Energy Consumption

An energy audit was performed to obtain and compare real and simulated energy use of the hotel. Energy use by hotels is influenced by their specific operational features, which include operating schedules for the different and number of functional facilities, services offered, fluctuation in occupancy levels daily and seasonally, and variations in customer preference relevant to indoor comfort. All facilities were considered in the audit because the actual energy use of the hotel was derived from energy bills. The current energy use, derived from energy bills, was extrapolated for the year 2016. 

During the energy audit, the authors collected all information about the building’s physical (frame, plants, etc.) and operational (occupancy, equipment data sheets, etc.) features, which allowed for the modelling of the building using software for energy simulation. In case of unknown operational details, reference was made to Italian standards. The energy performance (EP) index of the building was calculated using a semi-steady-state software (Thermus). It allows a tailored rating energy assessment according to the indications of the UNI CEI EN 16247-2 standard to be carried out, which takes into account the real users’ conditions (e.g., occupancy, actual consumption of hot water, ventilation flow rates, etc.). The tailored rating energy evaluation, as described in the technical specification UNI/TS 11300, and UNI EN 15603:2008 “Energy performance of buildings—overall energy use and definition of energy ratings” is aimed at assessing an existing building with an acceptable energy performance error, adapting to the real usage profile that can be concretely compared with the results that arise, for example, from the reading of energy bills. Once the model was created, all the solutions analyzed represent a true "simulation" of the actual savings that can derive from the energy saving solutions.

The energy performance was expressed by the annual non-renewable primary energy consumption for heating, DHW preparation, and lighting needed for standard building operation. The result of the calculation of energy performance of the building in its current state was primary global energy not renewable, EP_gl,nren_ = 310.95 kWh·m^−2^·y^−1^, and primary global energy renewable EP_gl,rin_ = 97.10 kWh·m^−2^·y^−1^ with a C energy class level.

Table 1 reports the real and simulated data referring to electricity and gas consumption. The average electricity consumptions in 2016 were about 48.45 kWh, while the gas consumptions were about 64.68 kWh. These data were used to verify and validate the model.

### 3.4. Proposed Retrofit Strategies

**Figure 3:**
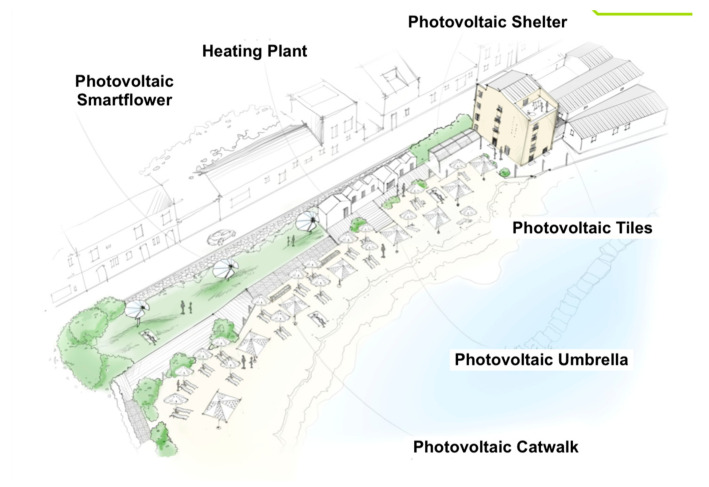
Sketch of the interventions.

The proposed energy retrofit strategies aim at improving the building energy class, ensuring high return of the investment over the short-term and the significant reduction of greenhouse gas (GHG) emissions. Starting from the analysis of the current building performance, energy efficiency measures were addressed to improve it [38,39]. The energy efficiency measures chosen for energy retrofitting are reported in Figure 3 and Table 2.

The selection of the technical solutions proposed took into account: The specific characteristics of the historic hotel building (site and orientation, energy consumption, seasonality, etc.); the expected energy performances; the environmental performances; and the economic–financial feasibility. The solutions were in compliance with the regulations of Legislative Decree no. 42 of 22 January 2004 (Code of Cultural Heritage and Landscape) [40,41,42]. For example, the hotel façades have high cultural value and must be preserved [43,44]. Therefore, the only choice was to insulate the walls from the inside.

## 4. Results

### 4.1. Energy Performances Results

Several numerical analyses were carried out considering the energy efficiency measures described in the previous section, in order to verify the effectiveness of such measures to make the hotel meet the target performance. The calculations were performed with a national certified software (Thermus) by CTI (Italian Thermo-Technical Committee), based on relevant national and international standards. The software allows for the calculation of the final and primary energy and demanded carbon emission levels for all energy services, namely space heating and cooling, domestic hot water, and lighting. In Table 3 the energy efficiency measures of the nine interventions compared to the base case (BC) are reported.

It can be noticed that the interventions on the energy systems were more effective if compared to those on the frame, despite the latter avoiding thermal bridges and improving indoor thermal comfort. In particular, the highest performances were achieved by means of the installation of a water-to-water HP.

### 4.2. Economic Performances Results

The expected energy performances was supposed point and continuous revenues and costs. Within the time span T the annual cash flow was calculated as the difference between annual revenue and cost, based on which the investment profitability can be valuated [45]. The annual cash flows include:

- Revenues, concerning savings, S, due to the improvement of the energy performances; government subsidies, s;
- Global costs, concerning initial expenses for refurbishment works instalment $I_{0}$; additional expenses for professional services, p; value-added tax for both the above-mentioned items, t; annual operating cost for each energy service, m; discounted annual sinking funds f for replacing the systems after their usable life l, regarding the total amount of the above listed items; the annuity for system replacement is:
  (1) $$f=I_{o}\frac{r}{{\left(1+r\right)}^{l}-1}$$

Then:

(2) $$NPV=-I_{0}+\sum_{i=1}^{T}\frac{\left(S+s-p-t-m-f\right)}{{\left(1+r\right)}^{i}},$$

(3) $$IRR=r_{\left(NPV=0\right)},$$

(4) $$DPbP=T_{\left(NPV=0\right)},$$

Table 4 displays the values of these indexes for each solution and for the sum of all solutions.

Table 5 lists the standard scores $K_{T}$ and $K_{E}$ for r=5%; the weighted average score $K_{T}$ for T; the resulting ranking of the nine solutions; and the economic result and the financial indexes.

According to the $K_{T}$-ranking of the solutions, nine strategies were arranged including, progressively, the best solutions (marked with 1 in Table 6), starting from the heat pump system (Solution 3). The ninth strategy comprises all solutions.

The arrangement of the elementary solutions in different strategies is a necessary simplification proposed in such a preliminary experiment aimed at testing the proposed pattern. A future insight will be aimed at taking into account the inter-dependence of the solutions with the different results in terms of energy, environmental, and economic–financial performances. 

The nine strategies were finally compared in economic–financial terms, as displayed in Table 7, also reporting the standard scores with the weights of the three performances, the weighted average score (WAS) $K_{E}$, and the resulting ranking.

**Figure 4:**
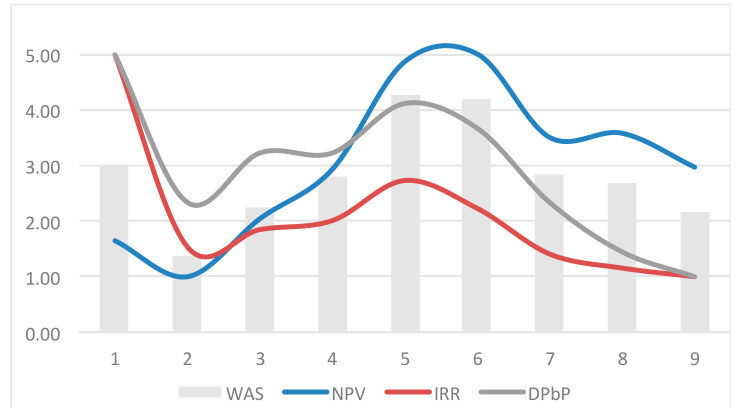
Set of strategies showing the economic–financial performances and the related ranking of solutions.

**Figure 5:**
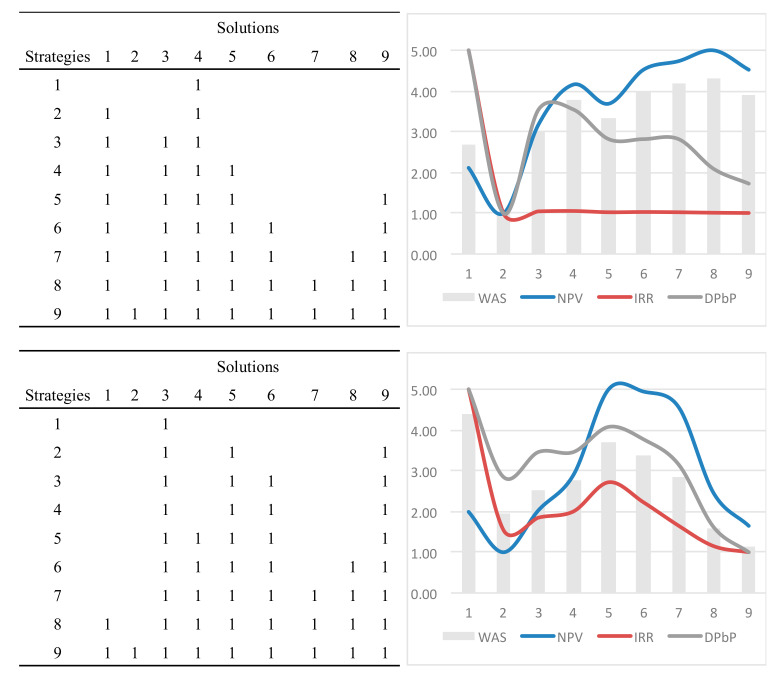
Scenario analysis: Comparison of two related strategies.

The model allows us to provide a top-down ranking of the solutions to be implemented according to the overall technical–environmental performance. Successively, the model has allowed us to select the optimal strategy, that is the one maximizing the economic–financial performance excluding the less profitable solutions. The process is synthetically represented in Figure 4, which displays the results of Table 7; here it is possible to highlight the increase of NPV up to strategy 6, beyond which it definitely decreases due to less profitable solutions, such as thermal insulation and window replacement. The strategic weight system can be arranged in different ways in order to provide developers and public authorities with further information for the best choice according to specific environmental, economic–financial, and technical issues. Two further scenarios were simulated (Table 8) and compared (Figure 5).

Three different scenarios have been identified, each of which is characterized by a different weight system ($\lambda_{T1}$ (EP_gl,nr_), $\lambda_{T2}$ (EP_gl,ren_), $\lambda_{T3}$ (EC), $\lambda_{T4}$ (CO_2_), $\lambda_{E1}$ (NPV), $\lambda_{E2}$ (IRR), $\lambda_{E3}$ (DPbP)) expressed by the decision makers, as shown in Table 8.

The three scenarios were arranged as follows: Scenario 1 outlines an intermediate approach to energy and environment; the decision makers, in this case, consider the environmental criteria (energy class and CO2 emissions) just a little more important than the ones related to the energy sources; the economic criteria (NPV) is considered more important than the financial ones (internal rate of return and discounted payback period), and considers a moderate discount rate (r=5%). Scenario 2 outlines a profile mainly aimed at sustainability, as shown by the primary interest in renewable energy sources and in the energy class, as for technical environmental criteria; furthermore, it is characterized by the modest interest for the financial criteria, and above all as for the very low discount rate (r=2%). Scenario 3 outlines a typical entrepreneurial profile mostly aimed at the private interest, and is the opposite of Scenario 2, given that the correspondent weights are inverted, and the discount rate very high. Therefore, two further scenarios were simulated and compared in Figure 5. Such overall assessment approach allows us to outline the nZEB profile of each strategy. For example, scenario 2 shows that the best nZEBs solution (Strategy 8) is the most profitable (Figure 5a,b); on the contrary, scenario 3 shows that the most profitable strategy (Strategy 1) is the worst solution to reach the nZEBs target. As a result, scenario analysis can be a useful tool for the choice of the best strategy, not only for the stakeholders, but for the stockholders as well: Scenario 1 is the best trade-off for entrepreneurial investments; scenario 2 considers investment with a shorter payback period and it is attractive for private investors; scenario 3 is particularly suitable for social and environmental issues and is mostly encouraged by public (or environmental) authorities [46].

## 5. Discussions and Conclusions

Overall, conclusive remarks can be synthesized as follows. For obtaining a nearly zero-energy balance, it is necessary to upgrade the performance of the building envelope in combination with the energy generation system. The analyzed energy efficient measures under-investigated are that one commonly used in the current building design. However, only such strategy, which significantly contributes to diminishing the energy demand of the building, is not sufficient to reach a nearly zero balance of the energy needs. Therefore, the local production of energy through renewable sources is compulsory, which would allow for the obtainment of a nearly zero-energy balance. Due to the climatic zone, frame energy retrofit solutions such as thermal insulation, window replacement, and so on, are not economically significant. The results of this paper highlight that the Italian transposition of the nZEB concept, and the constraints of historic buildings during the renovation process, reduces the choice of technologies useful for the building refurbishment towards the nearly zero-energy target. However, the heat pump associated with a PV system seems to be the most effective solution that could be able to improve indoor comfort conditions for the guests. 

Combining the thermo-technical approach with the economic-financial issues, and taken into account also the environmental performance of the envisaged solutions, a wider assessment prospect arises outlining the different and differently motivated decisional profiles [46,47].

Such decisional profiles have been outlined by the three scenarios, each of them defined by a specific weight system differently privileging the technical, the environmental and the economic-financial issues.

The first scenario, that can be considered the normal one due to the roughly equal importance attributed to the three issues, shows a significant preference for strategies 5 (assessed as the fairest) and 6 (slightly less preferable).

A significantly environment-oriented scenario, mostly involving the stakeholders, suggests the completion of the overall nZEBs plan as proposed in all its works and installments, considering as preferable strategy 8 (supposing the implementation of almost all the works), then 7 and 9 (the latter supposing the implementation of all the works).

The last scenario, mostly focused on the economic-financial issues, mainly involving the stockholders, paradoxically suggest as preferable strategy 1, including just the heat system replacement, so excluding any other work concerning the environmental sustainability, the personal comfort increase and the enhancement of the energy performance of the building, then reducing any its development potential. As a general perspective, we remark that the whole nZEBs plan [48] here proposed reaches significant economic-financial performances, so that the stockholders can be supposed to implement it 

The nZEBs approach has been developed, so far, in many ways, mostly in the technical building context and from the point of view of economic profitability. The experience we carried out aims at combining the two above issues (technical and economic) in a valuation-programming model able to support both private developers and public administrations throughout the negotiation path, involving private investors committed in urban transformation processes affecting free areas and/or existing buildings. Usually, the public–private interaction consists in the payment of a permit fee by the developer to the public administration. Such fees aim at somehow balancing private interest (gained) and public value (lost) and can be increased or reduced in order to discourage or encourage the transformation. The new approach can become a useful decisional and management tool for the public administration in order to choose the best strategy to reach the nZEBs standard. 

Energy use by hotels is also influenced by variations in customer preference relevant to indoor comfort. This study can be a useful tool to change the perception by hotels manager that high energy use is necessary to ensure the comfort of guests. Moreover, the model can be also used considering the fee of building permits and solutions for the private sector that are not profitable but are environmentally friendly for the public. The hotels can use the tool for the choice of the best strategy in order to reduce operational costs and CO_2_ emissions, improve image, comfort, and services for the guests.

## Figures and Tables

**Table 1:** Real and calculated energy delivered.

Source	Real	Simulated	Variation
Natural Gas	5910 Sm^3^	5138 Sm^3^	13.0%
	64.68 kWh_th_	56.23 kWh_th_	
	150 kWh·m^−2^·y^−1^	131 kWh·m^−2^·y^−1^	
Electricity	48.45 kWh	42.52 kWh	-
	113.0 kWh·m^−2^·y^−1^	99.0 kWh·m^−2^·y^−1^	

**Table 2:** Interventions and proposed solutions.

N	Energy Efficiency Measures	Proposed Solutions
1	Envelope Thermal Insulation (Internal)	Vacuum Insulation Panel
		s = 10 mm; k = 0.005 W/m·K
2	Windows Replacement	Double Pane Wood Windows
		U = 1.1 W/m^2^·K
3	Energy Systems Replacement	Water-To-Water Polyvalent HP for Space Heating, Cooling and DHW Heating Power = 21 kW; Cooling Power = 19 kW; COP = 4.52
4	Light Bulbs Replacement	LED
5	Renewable Energy System	Photovoltaic Tiles; Area = 89.0 m^2^, P = 11.60 kWp
6		Photovoltaic Shelter; Area = 65.0 m^2^, P = 10.50 kWp
7		Photovoltaic Smartflower; Number = 3, P = 6.93 kWp
8		Photovoltaic Catwalk; Area = 158.0 m^2^, P = 9.50 kWp
9		Photovoltaic Umbrella; Area = 330.0 m^2^, P = 19.80 kWp

**Table 3:** Energy efficiency measures of the nine interventions.

	B	1	2	3	4	5	6	7	8	9
$T_{1}$: EP_gl,nr_ kW·m^−2^·y^−1^	311	303	296	130	220	239	248	270	254	195
$T_{2}$: EP_gl,rin_ kW·m^−2^·y^−1^	97	54	87	126	62	102	101	95	99	114
$T_{3}$: CO_2_ Kg·m^−2^·y^−1^	65	64	62	29	45	49	51	56	52	39
$T_{4}$: Energy Class	**C**	**B**	**B**	**A3**	**C**	**A1**	**A1**	**B**	**B**	**A2**

**Table 4:** Economic analysis results for each solution.

		Solutions									TOT
		1	2	3	4	5	6	7	8	9	
NPV	*€*	−44.2	−17.9	69.4	57.1	30.7	26.1	2.2	4.1	−19.3	**108.1**
IRR	*%*	−31	−31	31	684	15	13	−10	−8	−17	**9.2**
DPbP	*y*	-	-	3	2	5	5	23	18	-	**12**

**Table 5:** Technical environmental and economic–financial performance scores and *T*-ranking of the nine solutions.

	Solutions									Weights
	1	2	3	4	5	6	7	8	9	
EP_gl,nr_	1.0	1.2	5.0	2.9	2.5	2.3	1.8	2.1	3.5	0.2
EP_gl,rin_	5.0	3.2	1.0	4.6	2.3	2.4	2.7	2.5	1.7	0.2
CO_2_	1.0	1.2	5.0	3.2	2.7	2.5	1.9	2.4	3.9	0.3
Class	2.0	2.0	3.0	1.0	5.0	5.0	2.0	2.0	4.0	0.3
$K_{T}$	2.1	1.8	3.6	2.7	3.3	3.2	2.1	2.2	3.4	-
Ranking	7	9	1	5	3	4	8	6	2	-
NPV	1.0	1.9	5.0	4.6	3.6	3.5	2.6	2.7	1.9	-
IRR	1.0	1.0	1.3	5.0	1.3	1.2	1.1	1.1	1.1	-
DPbP	1.0	1.0	4.8	5.0	4.5	4.5	1.3	2.2	1.0	-

**Table 6:** Strategies for elementary solutions.

Strategies	Solutions								
	1	2	3	4	5	6	7	8	9
1			1						
2			1						1
3			1		1				1
4			1		1	1			1
5			1	1	1	1			1
6			1	1	1	1		1	1
7	1		1	1	1	1		1	1
8	1		1	1	1	1	1	1	1
9	1	1	1	1	1	1	1	1	1

**Table 7:** Economic–financial results for the nine strategies in real valuations and standard scores.

	Strategies									Weights
	1	2	3	4	5	6	7	8	9	
NPV	69.4	50.1	80.8	106.9	164.0	168.1	123.9	126.1	108.1	-
IRR	36%	13%	15%	16%	21%	17%	12%	10%	9%	-
DPbP	3.0	9.0	7.0	7.0	5.0	6.0	9.0	11.0	12.0	-
NPV	1.7	1.0	2.0	2.9	4.9	5.0	3.5	3.6	3.0	0.6
IRR	5.0	1.5	1.8	2.0	2.7	2.2	1.4	1.2	1.0	0.2
DPbP	5.0	2.3	3.2	3.2	4.1	3.7	2.3	1.4	1.0	0.2
$K_{E}$	3.0	1.4	2.2	2.8	4.3	4.2	2.8	2.7	2.2	-
Ranking	3	9	7	5	1	2	4	6	8	-

**Table 8:** Strategic variables for alternative scenarios.

	Technical–Environmental Profile				Economic–Financial Profile			
Scenario	$\lambda_{T1}\;({\mathrm{EP}}_{\mathrm{gl},\mathrm{nr}})$	$\lambda_{T2}\;({\mathrm{EP}}_{\mathrm{gl},\mathrm{ren}})$	$\lambda_{T3}\;(\mathrm{EC})$	$\lambda_{T4}\;({\mathrm{CO}}_{2})$	$\lambda_{E1}\;(\mathrm{NPV})$	$\lambda_{E2}\;(\mathrm{IRR})$	$\lambda_{E3}\;(\mathrm{DPbP})$	r
1	0.20	0.20	0.30	0.30	0.60	0.20	0.20	5%
2	0.10	0.45	0.30	0.15	0.80	0.10	0.10	2%
3	0.45	0.10	0.15	0.30	0.20	0.40	0.40	8%
